# Etiology, clinical presentation, and outcome of children with fulminant hepatic failure: Experience from a tertiary center in Pakistan

**DOI:** 10.12669/pjms.36.6.2375

**Published:** 2020

**Authors:** Sidra Talat, Sabeen Abid Khan, Nismat Javed, Munir Iqbal Malik

**Affiliations:** 1 Sidra Talat, MBBS, FCPS. Senior Registrar, Department of Paediatrics, Shifa College of Medicine, Shifa Tameer-e-Millat University, Islamabad, Pakistan; 2 Sabeen Abid Khan, MBBS, FCPS. Assistant Professor, Department of Paediatrics, Shifa College of Medicine, Shifa Tameer-e-Millat University, Islamabad, Pakistan; 3 Nismat Javed, Final year medical student, Department of Paediatrics, Shifa College of Medicine, Shifa Tameer-e-Millat University, Islamabad, Pakistan; 4 Munir Iqbal Malik, MBBS, DABP, MD. Professor and Paediatric Gastroenterologist, Department of Paediatrics, Shifa College of Medicine, Shifa Tameer-e-Millat University, Islamabad, Pakistan

**Keywords:** Fulminant hepatic failure, Children, Pakistani, Overlapping etiologies

## Abstract

**Objectives:**

To determine etiologies, clinical presentations and outcomes of children with fulminant hepatic failure in the first liver transplant center of Pakistan.

**Methods:**

It was a retrospective, observational study, conducted in Paediatric Gastroenterology Department of Shifa International Hospital. Patients between one month to 16 years were included who fulfilled the Pediatric Acute Liver Failure study group (PALFSG) definition of acute liver failure as biochemical evidence of liver injury with no known co-existing chronic liver disease, coagulopathy not corrected by vitamin K, an International Normalized Ratio (INR) greater than 1.5 if the patient has encephalopathy, or greater than 2.0 if the patient does not have encephalopathy. The data collected was recorded on a self-constructed proforma after IRB approval.

**Results:**

There were 28 patients in the study which ncluded 17 males and 11 females with a mean age of 72.86±52.50 months. The most common etiologies were Hepatitis A (29%) in isolation or co-infection with Wilson Disease, typhoid fever. It was followed by seronegative hepatitis (29%). Majority (64%) had acute presentation (7 to 28 days), jaundice (82%) being the most common symptom. Severity of encephalopathy was significantly associated with outcome (p=0.02). There were 6 (21%) patients who succumbed to death.

**Conclusions:**

The study highlights infective diseases as the predominant etiology causing fulminant liver failure in children. Our study highlights lower mortality in children

## INTRODUCTION

Fulminant hepatic failure refers to a highly specific and fatal albeit rare clinical syndrome, manifesting as various abnormalities in liver function tests in a patient without pre-existing hepatic pathology. The deterioration in liver function is associated with coagulopathy and altered level of consciousness due to hepatic encephalopathy.^1^ However, owing to the difficulty in the assessment of hepatic encephalopathy in young children and infants, presence of signs of encephalopathy is not a part of diagnostic criteria in children as opposed to adults.^2^,^3^ There is wide variation in the etiology leading to fulminant hepatic failure depending on the age group and geographical location, with the acute viral hepatitis^4^ and drugs among the leading causes.^5^ Other causes leading to fulminant hepatic failure include seronegative hepatitis, metabolic liver diseases, autoimmune hepatitis, hemophagocytic lymphohistiocytosis and sepsis. Mortality associated with this devastating event is reported to be between 50^6^ and 70 percent.^7^

Supportive treatment becomes less effective with advanced levels of coagulopathy and coma. Liver transplantation remains the only possible solution in these patients. The need to pick subtle signs and laboratory parameters pointing to a bad prognosis, the importance of early start of effective treatment and fewer management options for advanced cases calls for a standardized diagnostic and management strategy. Delayed referrals to centers equipped with the facility of hepatic transplantation is a major cause of increased mortality in these patients. It is particularly relevant in a country like Pakistan where, facilities catering the need for liver transplantation are also inadequate meet the need of the masses and concept of multidisciplinary team approach is yet developing.^8^

This study was conducted at the first Paediatric liver transplant center in Pakistan to investigate etiology, clinical presentation, and outcome after the diagnosis of fulminant hepatic failure was established.

## METHODS

This observational retrospective study was conducted in Paediatric department of Shifa International Hospital. The hospital serves as a tertiary care hospital with services of Paediatric gastroenterology and Paediatric liver transplant. All patients who were referred to us between 2012 to 2019 aged one month to 16 years who fulfilled the criteria for fulminant hepatic failure (FHF) were included in the study.

Patients with chronic liver diseases as suggested by history of symptoms more than six months and incomplete files were excluded from the study.^9^ Pediatric Acute Liver Failure study groups (PALFSG) consensus definition of acute liver failure in children was used as inclusion criteria which states biochemical injury of liver injury with no known co-existing chronic liver disease, coagulopathy not corrected by vitamin K, an International Normalized Ratio (INR) greater than 1.5 if the patient has encephalopathy, or greater than 2.0 if the patient does not have encephalopathy.^10^ In children, the emphasis is on presence of significant coagulopathy in the absence of sepsis.^11^ FHF was further classified according to hyper-acute (less than seven days), acute (8 to 28 days) and subacute presentations (5 to 12 weeks) depending on time taken to develop encephalopathy after onset of jaundice.

Data including age, gender, height, weight, liver function tests like alanine aminotransferase (ALT, Total bilirubin, direct bilirubin, alkaline phosphatase, coagulation profile prothrombin time (PT/INR), serum albumin and blood sugar levels were studied from the case files. Laboratory workup pertaining to specific etiologies like viral markers, serum ceruloplasmin, 24-hour urinary copper levels, metabolic workup, autoimmune liver profile, blood cultures and septic workup was also reviewed for each patient. Clinical presentation, grade of encephalopathy, time from first symptom to encephalopathy and etiology was taken into consideration. Patient’s outcome in terms of survival on supportive care, survival after liver transplant and/or death was recorded from patients file. Information was taken on self-designed questionnaire. (Ref: IRB # 1157-433-2018, Dated: October 23, 2018) Institutional review board approval was taken. Data was reviewed on SPSS 23. Chi-square test was applied and a p value of <0.05 was taken as statistically significant.

## RESULTS

There was a total of 28 patients, 17 males (61%) and 11 females (39%) included in the study who fulfilled the inclusion criteria. The mean age of the participants was 72.86±52.50 months. The mean height was 97.95±32.82 cm and mean weight was 19.09±11.12 kg. The various etiologies of hepatic failure are shown in Table-I.

**Table-I:** Etiologies of Fulminant Hepatic Failure.

Etiologies of Hepatic Failure	Percentage
Septicemia	7%
Hepatitis A and known G6PD	3%
Valproate Toxicity	4%
Salmonella Sepsis	3%
CMV	4%
Seronegative Hepatitis	29%
Dicarboxylic Aciduria	3%
Wilson’s	4%
Hepatitis A	29%
Hepatitis A and Typhoid	3%
ALL Relapse	4%
Hepatitis A and Wilson’s	7%

The participants underwent a series of investigations. The maximum and minimum values of baseline liver function tests, random blood glucose and ammonia were also recorded and are shown in Table-II.

**Table-II:** Laboratory Workup and their Ranges.

Lab Investigations	Mean	Minimum	Maximum
ALT (U/L)	1173.00	8.00	4828.00
AST(U/L)	1070.00	124.00	6370.00
GGT(U/L)	175.00	34.00	1174.00
ALP (U/L)	361.00	51.00	1588.00
PT(Sec)	36.00	10.00	124.00
INR	3.33	1.00	9.90
Total Bilirubin (mg/dl)	25.73	0.95	63.00
Indirect Bilirubin(mg/dl)	8.35	0.30	22.00
Direct Bilirubin(mg/dl)	17.60	0.36	57.50
Albumin (gm/l)	2.84	1.90	4.20
BSR(mg/dl)	99.00	35.00	232.00
Ammonia (mg/dl)	204.00	65.00	564.00

The mean prothrombin time was 36.93±30.52, INR was 3.33±2.33, total bilirubin 25.73±18.06, direct bilirubin was 17.61±14.14 mg/dl, mean albumin 2.84±0.53 g/l, alkaline phosphatase levels of 361.54±308.44 U/L. Mean ammonia levels were 204.41±109.57 mg/dl and blood sugar levels were 99.69±47.21 mg/dl. In the electrolytes, serum sodium was 125.95±34.23 mEq/l, serum potassium 3.75±1.20 mEq/l, bicarbonate levels were 18.85±4.86 mEq/l and phosphorus was 3.26±1.94 mEq/l. Complete blood count profile of patients showed mean hemoglobin 9.87±1.79 g/dl, total leukocyte counts of 20082.14±14113.38, mean platelet count was 285188.68±195826.72.

The most common (64%) type of presentation was acute which ranges from seven to 28 days. This was followed by hyper acute presentation (22%) which was less than seven days and lastly, sub-acute presentation which presented in 5-12 weeks (14%). The most common presenting symptom was jaundice (82%). The outcome of all the participants was also recorded. The outcome was divided into four categories: six patients (21%) Left against Medical Advice (LAMA), one patient (4%) was discharged on Request (DOR), 15 (54%) patients survived while six patients (21%) succumbed to death. Amongst the 15 patients who had survived, 14 survived on supportive care and one patient underwent living donor liver transplant. The etiologies for patients who had died were also recorded. The most common etiology was Hepatitis A (33%). The other etiologies included Salmonella sepsis (17%), seronegative hepatitis (17%), malignancy (17%) and Hepatitis A co-infection with Wilson’s disease (17%). The patients in hepatic failure presented with varying degrees of encephalopathy. These degrees have been shown in Table-III.

**Table-III:** Outcomes and Grades of Encephalopathy.

Outcome	Encephalopathy				

	Alert	Grade 1	Grade 2	Grade 3	Grade 4
LAMA	0	2	2	1	1
DOR	0	0	0	0	1
Survived	1	9	4	1	0
Death	0	0	1	2	3

P-Value = 0.02

The degrees of encephalopathy were significantly associated with outcomes (p= 0.02). Patient mortality was also related to the level of hepatic dysfunction particularly dysfunction in synthesis of essential clotting factors. INR more than 4 was seen in 9 patients and 19 had INR less than 4 however, it was not found to be significantly associated with outcome.

Chi-square test was also applied to determine if there were significant differences of outcomes as compared to levels of ammonia, bilirubin, albumin and ALT. They were all found to be non-significant.

## DISCUSSION

Fulminant hepatic failure is an uncommon but serious condition with high mortality reported worldwide. Our study highlights diverse causes of fulminant hepatic failure presenting in Pakistan from the first Paediatric liver transplant facility. Acute viral hepatitis A (29%) is the leading cause of fulminant hepatic failure seen in our study. Co-infection with Hepatitis A and Wilson disease was seen in 2 (7%) patient and hepatitis A co-infection with Typhoid fever was also seen in 1 (3%) patient. This finding highlights the fact that low threshold should be kept, and patients should be screened for other co-existing etiologies along with hepatitis A. Limited studies done in Pakistan have all shown Hepatitis A as the leading cause of FHF. Similarly data from neighboring countries like India shows same pattern, however, Hepatitis E is reported as the predominant cause in Indian adults.^12^ Amongst the other infective etiologies, we had two(7%) patients with Septicemia, one patient (3%) had salmonella sepsis with organism resistant to ceftriaxone. To our knowledge this was the first case of ceftriaxone resistant salmonella sepsis causing fulminant hepatic failure in a six-year-old child with a rapidly deteriorating clinical course leading to mortality.^13^ Previously Salmonella sensitive to ceftriaxone has been case reported as etiology for FHF in children from middle east.^14^ CMV hepatitis was seen in one patient (4%). Half of all the causes are infective in origin. The second most common cause in our study was sero negative (non A non E) hepatitis 8 (29%) patients. Studies from other centers in Lahore and Karachi has shown similar trends.^15^

Amongst the metabolic causes Dicarboxylic aciduria and Wilson disease was seen in one (3%) patient each. Drugs Valproate toxicity which was idiosyncratic was seen in one patient (4%) which is in contrast with western literature where acetaminophen toxicity is the most common cause.^10^ Among malignancy we had one patient with ALL relapse presenting with FHF. In our study 15(53.5%) patients survived, 14 improved on supportive care and only one patient with seronegative hepatitis survived following liver transplant.^16^ Timely liver transplant remains the only cure for survival in patients not improving on supportive care.^17^ Our rate of liver transplant is similar to other studies from resource limited countries, owing to delayed referrals, rapidly worsening disease process and limitation of the procedure as well.^18^ In contrast 10% of Paediatric liver transplants done in America are due to FHF.^19^ Mortality was seen in 6 (21.4%) patients. seven (25%) patients went LAMA/DOR so difficult to comment on their outcome. Mortality (21%) reported in our study is lower than another study reported from Lahore (36%).^20^ The degree of encephalopathy was significantly associated with mortality (0.02). This trend has been observed in other studies as well.^21^ Prothrombin time and INR were not found to be significantly associated with outcome in our study, a similar trend has been observed in some studies.^22^

The study shows infective etiologies as the predominant cause of fulminant hepatitis in Pakistani children, and highlights the need for improved sanitation, water supply, hand and food hygiene, vaccination^23^ and awareness.

### Limitations to the study

here were few patients who were available as participants in the study. The sample size was also small due to records of patients being incomplete. Additionally, the study is retrospective in nature which is another limitation.

## CONCLUSION

The study highlights infective diseases as the predominant etiology causing fulminant liver failure in children. Our study highlights lower mortality in children, majority of patients improved with supportive care while one patient underwent liver transplant.

### Authors’ Contributions:

**ST:** Conception of study, data collection.

**SAK:** Drafting of study, interpretation of results.

**NJ:** Data collection & analysis and is responsible for integrity of research.

**MIM:** Final revision, drafting, expert opinion.
